# A Review on Strategies to Manage Physician Burnout

**DOI:** 10.7759/cureus.4805

**Published:** 2019-06-03

**Authors:** Rikinkumar S Patel, Shiana Sekhri, Narmada Neerja Bhimanadham, Sundus Imran, Sadaf Hossain

**Affiliations:** 1 Psychiatry, Griffin Memorial Hospital, Norman, USA; 2 Psychiatry, Adesh Institute of Medical Sciences and Research, Bathinda, IND; 3 Public Administration / Health Care Administration, Drake University, Des Moines, USA; 4 Neurology, Indiana University School of Medicine, Indianapolis, USA; 5 Psychiatry, Brookdale Hospital, Brooklyn, USA

**Keywords:** burnout, depression, physician burnout, physician depression, emotional exhaustion, management

## Abstract

Physician burnout is an emerging condition that can adversely affect the performance of modern-day medicine. Its three domains are emotional exhaustion, depersonalization, and a sense of reduced accomplishment among physicians, with the Maslach Burnout Inventory (MBI) being the gold standard questionnaire used to scale physician burnout. This concern not only impacts physicians but the entire healthcare system in general. There is growing awareness regarding the mental health of physicians and the consequences faced by the healthcare system as a result of burnout. According to a recent study, more than 50% of physicians reported suffering from at least one burnout symptom. In this review article, we aim to identify the causes leading to burnout, its impact on physicians, and hospital management as well as interventions to reduce this work-related syndrome. Some contributing factors leading to burnout are poor working conditions with long work shifts, stressful on-call duties, lack of appreciation, and poor social interactions. Burnout can lead to adverse consequences, such as depression, substance use, and suicidal ideation in physicians and residents. This can result in poor patient care increasing total length of stay, re-admissions, and major medical errors. Due to increased scrutiny of patient and healthcare costs, along with increased lawsuits as a result of major medical errors, it is crucial for both the hospital management and physicians to recognize and address burnout among physicians. Comprehensive professional training such as Cognitive behavioral therapy (CBT), stress-reducing activities such as mindfulness and group activities, and strict implementation of work-hour limitations recommended by Accreditation Council for Graduate Medical Education (ACGME) for residents are a few methods that may help to manage burnout and increase productivity in hospitals.

## Introduction and background

Burnout is a psychological syndrome that can be defined as “emotional and exhausting conditions related to working environment” [1]. The three domains of burnout are emotional exhaustion, depersonalization, and low personal achievement, and these seem to be more prevalent in the ‘helping’ profession, such as physicians and teachers [1-2]. Physician burnout has reached epidemic levels, with the prevalence of near or exceeding 50% among physicians in training (medical students, residents) as well as practicing physicians in the US [3]. A nationwide survey conducted in 2014 by Shanafelt et al. evaluated the prevalence of physician burnout and found that 54.4% of US physicians reported at least one symptom of burnout as compared to 45.5% in 2011, along with a decline in satisfaction with work-life balance from 2011 to 2014 (48.5% vs. 40.9%) [4]. Patel et al. [5] revealed the factors associated with physician burnout and it's consequences affecting a physician's overall health, and patient outcomes and healthcare system. This represents a public health crisis with negative impacts on patient care, professionalism, physician’s care and safety (including various issues such as mental health concerns and motor vehicle accidents), and healthcare systems and organizations [3].

Physician burnout usually arises from work-related stressors, which can be due to multiple factors such as excessive workload and hours, clerical burden, electronic medical records (EMR), inefficient work process, complex patient-related decisions, poor work-life integration, hostile working environment, and organizational factors (misaligned organizational culture, values, and leadership behaviors) [6-7]. Burnout among physicians has been associated with a significantly higher risk of making errors (e.g., medication errors, diagnostic errors, and decision-making errors, etc.), leading to sub-optimal patient care and lowering patient’s satisfaction with received medical care and adherence to treatment plans [8]. Furthermore, physician distress contributes to increased physician turnover, reduced productivity, job dissatisfaction, higher absenteeism, difficulties in interpersonal relationships (family and friends), and early retirement [7-9].

This is a global problem and has to be addressed immediately to prevent harmful effects on patient care and the failure of healthcare systems. Reducing individual and organizational risk factors and working on strategies to manage workloads and improve physician’s well-being can effectively decrease burnout. A study carried in the department of medicine at Mayo Clinic in Rochester, Minnesota showed a statistically significant decrease in the rates of depersonalization after interventional measures were introduced [10].

A literature search was conducted in our review study on MEDLINE, and EMBASE, from the years January 1, 1980 to December 1, 2018 using the keywords “burnout”, “physicians”, “doctors”, “depression”, “causes”, “emotional exhaustion”, “strategies to mitigate”, “patient care”, “healthcare organizations”, “consequences”, “symptoms”, “interventions”, and “treatment”. Publications found through this indexed search were reviewed and manually screened to identify relevant studies, including longitudinal case-control studies, cross-sectional studies, cohort studies, and systematic reviews.

In this review article, we aim to describe tools used for measuring burnout, factors leading to burnout and their negative consequences, and the importance of addressing burnout in physicians. We further propose strategies that can be used to prevent and reduce burnout. These are interventions directed at both individual and institutional levels and include cognitive behavioral therapy, mindfulness techniques, team-based interventions, group discussions, professional coaching, work-hour limitation, mental health training programs, and improving the work environment.

## Review

Measuring physician burnout

There are various survey tools available to measure physician burnout. According to a recent systematic review, the Maslach Burnout Inventory (MBI) is the most commonly used tool in scientific literature to measure burnout [10]. Other subscales of MBI include Burnout Measure (BM) and Shirom-Melamed Burnout Measure (SMBM), both of which consider exhaustion as the core measurement of physician burnout [11]. SMBM is a 22-item tool that measures personal fatigue, cognitive weariness, and emotional exhaustion [10]. BM is a 21-item tool measured on a seven-point scale determining the level of a person’s emotional, physical, and mental exhaustion [12]. 

Copenhagen Burnout Inventory (CBI), is a 19-item scale which measures burnout based on three criteria: work-related exhaustion, client burnout, and staff-related burnout, and is commonly used in Europe [11]. Oldenburg Burnout Inventory (OLBI) measures burnout in two parts-exhaustion and disengagement. Bergen Burnout Inventory (BBI) examines exhaustion, occupational inadequacy, and cynicism about the significance of work. Recently, a newly presented measurement survey module has been introduced which is known as Physician Burnout Questionnaire (PhBQ) and measures three main components of physician burnout: exhaustion, disengagement, and loss of expectations [11]. 

 MBI is a 22-item gold standard self-reported questionnaire used to measure burnout. Maslach first developed it in 1981, and this is a widely used tool. Although MBI is a known standard, it has also been criticized in various ways, one being its structure. MBI mainly measures three components: exhaustion, depersonalization/cynicism, and personal accomplishment/efficacy, but it ignores other components of physician burnout syndrome such as the meaning of work and loss of resources. Another restriction of MBI is its heavy focus on the emotional process instead of behavioral and cognitive aspects [11].

Strategies to manage physician burnout

The first few steps towards managing physician burnout are recognizing and measuring it, which include timely measuring physician well-being, proactively assessing and mitigating the regulatory burden on them, and allowing physicians to work on top of their license. These steps are implemented at Mayo clinic, which annually measures and compares their data with the national data enabling them to assess the workload and satisfaction levels of their physicians. It is further extending to inquire which department physicians are under stress of burnout and measures are taken accordingly to mitigate it [13-14].

Once burnout is recognized and measured, it is essential to take the initiative in addressing it. Interventions to reduce burnout can be classified into two main categories: physician-directed interventions focusing on individuals and organizational or structural directed interventions focusing on the work environment. Physician-directed interventions include mindfulness techniques, stress management techniques, and cognitive behavioral techniques to enhance job competence and improve communication skills and personal coping strategies. Organization-directed interventions can involve simple changes in the work schedule and environment, work tasks to reduce stress levels (e.g., reductions in the workload by improved team-work, changes in the work evaluation, increment of participation in decision-making, and supervision to minimize job demand and enhance job control), and more profound improvements in the operation of healthcare organizations. These interventions were associated with a meaningful reduction in emotional exhaustion and depersonalization scores and had more significant effects when compared with individual-directed interventions [3, 13, 15].

Additionally, some organizational and individual interventional programs have been very successful in reducing some of the factors contributing to physician burnout. A study conducted by Greden et al. [16] talks about a mental health training program in which a four-hour brief course known by the acronym RESPECT (Regular Contact is essential, Earlier the better, Supportive and empathetic communication, Practical help is not psychotherapy, Encourage help-seeking, Consider return to work options, Tell them door is always open and arrange next contact) lowered work-related sick leaves by 0.28 points. The program was designed for organizational managers to teach them how to recognize and prevent burnout. A study by Krasner et al. [17] reviewed an eight-week mindfulness course (2.5 hours/week) followed by a ten-month maintenance course and reported a reduction in burnout. The intervention was associated with short and long-term improvements in attitudes and well-being of patient-centered care. Another study by Rø et al. [18] demonstrated that counseling based on psychodynamic, cognitive, educational, and motivational interviewing (once a week) resulted in a reduction in emotional exhaustion.

Furthermore, in a physician influenced interventional study by Dunn P.M. et al. [19], from the years 2000 to 2005, physicians took three questionnaires (American College of Physicians Internal Medicine survey, MBI, and Quality Work Competency Survey). The organizational interventions were based on physician survey feedback and resulted in a decrease in emotional and work-related exhaustion. A study by Margalit, A.P. et al. [20] focused on bio-psychological teaching, didactic/interactive session, and Balint sessions, leading to a decrease in emotional exhaustion, depersonalization, and low personal accomplishment.

A family medicine program at Colorado University started, “APEX,” Ambulatory Excellence Program, in which medical assistants performed the initial workup including history taking, reconciling medications, scheduling visits, administrating vaccinations, and providing information regarding health education and preventive care. This helps the physician’s concentrate on physical examination and medical decisions. Though this method requires extensive training for medical assistants to work semi-independently following protocols, it turned out to be cost-neutral for the management. Also, burnout rates decreased from 53% to 13% in six months [14].

Working in a critical care setting is highly stressful due to high morbidity and mortality, patient overload, busy schedules, and daily confronting challenging ethical situations. All these factors attribute to the intensive care unit (ICU) physicians experiencing more than 50% of burnout syndrome [21]. A literature review by Marc Moss et al. [21] published different ways to alleviate burnout in ICU physicians. These included environmental interventions focusing on improving work atmosphere, practical communication training among physicians so they can structure work schedules with timely days off, team-based interventions targeting on team debriefings by using structured communication tools while holding team spirit and bonding together, and practitioner-based interventions centering assertive training, implementing relaxation techniques, mindfulness, healthy eating, exercising, resting well, and an overall work-life balance. Others include palliative care consultations, ethics consultations, establishing goals of care for each patient, and family counseling conference within 72 hours of ICU admission [21].

Providing professional coaching to trainees is another significant way to prevent burnout. This method has long been used by executives in corporate businesses and is making its way into the medical profession. Coaching is a creative process that enables doctors to maximize their professional potential and maximally utilize their mental capabilities and expertise to tackle life challenges. A core coaching principle is to increase a client’s internal sense of control, making a belief that one’s actions have as much or more impact on life outcomes than external forces or individuals [22-26]. Studies have indicated an inverse correlation between internal locus of control and burnout. A critical fact that is conveyed in professional coaching is that people have more control over their life circumstances and satisfaction than they typically realize. An essential principle of coaching is examining the clients’ fixed thoughts and negating their self-defeating inner dialogue by teaching them how to question automatic thoughts, beliefs, and perceptions, and thus differentiating between facts, assumptions, and interpretations. This is utilizing a practice in cognitive behavioral therapy which is extremely useful [27]. Coaching can increase efficacy and self-determination, which are vital factors to prevent burnout [28-29]. It also helps to strengthen professional skills, including decisiveness, time management, productivity, communication, leadership, and teamwork.

The Mindfulness-Based Stress Reduction (MBSR) program helps to reduce stress and manage emotions through mindfulness meditation. The University of California Center for Mindfulness states that “Mindfulness meditation is a quality which human beings already have, but they have usually not been advised that they have it, that it is valuable, or that it can be cultivated. Mindfulness is the awareness that is not thinking but which is aware of thinking, as well as aware of each of the other ways we experience the sensory world, i.e., seeing, hearing, tasting, smelling, feeling through the body.” It is an eight-week behavioral program and an educational course that offers meditation techniques (including mindful awareness of daily activities and communications), body scanning, and simple yoga postures. MBSR has been described as “a group program that focuses on the acquisition of mindful awareness of mindfulness” [30]. It is based on the following-non-judging, non-striving, acceptance, letting go, beginner’s mind, patience, trust, and non-centering [31, 32]. Evidence has shown that MBSR can mitigate stress [33-35], reduce burnout [17, 36], and improve empathy skills among medical professionals [17, 37].

Encouraging physician group curriculum with discussion groups incorporating elements of mindfulness, reflection, shared experiences, and small group learning has demonstrated to reduce burnout. Sharing experiences, emotions, and coping strategies to challenging situations, and relating with others can reduce burnout among physicians. Group activities like picnics, social outings, and charity work help build interpersonal relationships and team bonding. In a study, protected time (one hour of paid time every other week) for participants was provided by the institution to encourage these meetings. Empowerment and engagement at work can result in a feeling that work is meaningfully increased, and can reduce depersonalization, emotional exhaustion, and overall burnout. As the cause of burnout relates to ways physicians are trained during their residency period, it is of utmost importance to empower trainees to aim for physician wellness [9]. In a systematic review, it was shown that autonomy, the building of competence, strong social relatedness, sleep, and adequate time away from work, are essential factors for resident well-being. Perseverance is predictive of well-being, and greater well-being is associated with increased resident empathy [38].

Poor work conditions in primary care are associated with physician burnout and lower quality of care. The previously implemented triple aim by the Institute of Healthcare Improvement, to optimize health system performance (enhancing the patient experience, improving population health, and reducing costs), has been expanded to a quadruple aim, adding the goal of improving the work life of healthcare providers. Interventions, which worked, are mentioned below [39-41]:

(1) Scheduling monthly provider meetings focused on work life and personal challenges, as well as difficult patient care management issues.

(2) Offloading non-essential tasks to non-physician staff including hiring additional staff, having medical assistants (MA) enter patient data into the EMR, and consistently pairing MA’s with clinicians.

(3) Removing bottlenecks to care inpatient rooms regarding medication reconciliation, vaccinations and data entry.

(4) Reducing time pressure with plans to increase primary care visit appointment times.

(5) Developing a new prescription line to free up RN staff.

(6) Having clerks instead of physicians track forms and sending faxes.

(7) Using pre-visit planning and pre-appointment laboratory testing to reduce time wasted on the review and follow-up of laboratory results.

(8) Standardizing and synchronizing workflows for prescription refills, which can save physicians five hours per week while providing better care.

(9) Presenting OWL (web ontology language) data as a platform to discuss issues within the department, which ultimately improved communication.

Work-hour limitations (WHL) that have been imposed by ACGME in 2003 must be implemented strictly as they have shown to be effective. These limitations specify that resident physicians may work no more than 80 hours/week (averaged across four weeks) and no more than 24 consecutive hours (with six additional hours for transfer of care and educational activities), must have at least 24 following hours off per week and at least ten following hours off between shifts, and may take call no more frequently than one night in three [42]. A survey conducted at a University-based Internal Medicine residency in Seattle, Washington, showed an increase in the proportion of residents satisfied with their career and a decrease in the percentage meeting the criteria for emotional exhaustion. Overall, most residents (65%) approved of work-hour limitations [43]. In January 2017, National Academy of Medicine (NAM) along with Association of American Medical Colleges (AAMC) and the Accreditation Council for Graduate Medical Education (ACGME), established Action Collaborative on Clinician Well-being and Resilience. This collaboration comprises of more than 55 core organizations and network of more than 80 others including payers, researchers, government agencies, technology companies, patient organizations, trainees, and more. The main goals of this collaboration are to increase the prominence of clinician burnout, enhance organizations’ understanding of challenges to clinician well-being, propose more evidence-based solutions, and check their effectiveness timely [44]. The collaborative model will achieve these goals by promoting four different workgroups, to develop a resolution at the organizational, system and cultural levels. The “Research, Data, and Metrics” workgroup primarily collects validated assessments and evidence-based data based on which it identifies an indicator for measuring physician well-being. The “Conceptual Model” workgroup creates a comprehensive model, which establishes shared taxonomy based on important keywords. The “External Factors and Workflow” workgroup emphasizes on establishing novel team-based approaches for physician well-being. Lastly, “Messaging and Communications” workgroup locates key stakeholders and promotes evidence-based awareness and knowledge inspiring other organizations.

In the process of learning new methods to overcome physician burnout, “resilience” was given different definitions by different authors [45]. They all had the same agenda that building resilience means growing mentally stronger over the adversities bounce back stronger and maintain mental peace to avoid medical errors. Resilience is not static and is continually evolving, and it keeps the physicians to focus on their responsibilities with little or no room for errors [45]. The resilience scale is a 25-item self-questionnaire covering five domains of resilience: perseverance, self-reliance, purpose, authenticity, and patience. The other scale is the brief resilience scale to assess the resilience to bounce back challenges. Since there is no validated scale for measuring resilience, the ideal measuring scale is still on the way. These are the measures taken to assess resilience to check on one’s abilities when working in stressful environments and take appropriate steps to avoid consequences [45]. Due to chronic stress exposure, it leads to loss of top-down control of prefrontal cortex over the limbic system decreasing response to stress exposure. The hypo-activity of the prefrontal cortex also leads to reduced attention, working memory, and regulation of emotions, and inhibition of cravings [46]. Resilience research further explored that active coping mechanisms such as goal-directed behaviors, problem-solving, confronting problems, and seeking social support when required help reduce stress [46]. This avoids denial of the problem, illicit substance use, blaming someone else, and venting constant negative feelings. A recent survey done by Medscape National Report of Physician Burnout and Depression Report in 2018 recommended the top three strategies to reduce physician burnout would be increasing financial compensation which would in turn decrease stress related to expenditures, introducing flexible schedules, and decreasing government regulations [47].

The consequences of physician burnout also affect interpersonal relationships. In a study conducted among family practitioners-most of who were either married had a partner or were in live-in relationships (95.9%)-suggested ways to alleviate stress. This included encouraging flexible working schedules for physicians so that they can spend quality family time and participating in continued medical education (CME) where they have an opportunity not only to broadcast information but also feel supported from fellow members [48]. Addressing and catering to what individual physicians are passionate about is of utmost importance to promote career development and prevent burnout. Mayo Clinic researchers demonstrated that when academic physicians have less than 10%-20 % of their work time to do what they care about most, burnout rates increase to extremely prominent levels (>50 %). Hence it would be appropriate to provide at least one-half day per week for clinicians to do what they are most passionate about. Stanford’s Academic Biomedical Career Customization (ABCC) program focused on implementing career planning with transparent conversations around work-life needs and reducing work-life conflict by supporting grant editing, presentation preparation, and home task outsourcing services. Promoting career opportunities and advancement for part-time physicians with flexible career policies including part-time work and shared medical practices are critical for work-life balance [49]. A study of US physicians has shown those with the highest work-life balance have the lowest rates of burnout. It was seen that part-time physicians are satisfied with their jobs, provide top-quality care, and are less likely to leave their jobs. The gender differences in burnout identified among US physicians are not seen in the Netherlands, where 75% of women physicians work part-time. Part-time options allow institutions to use a more flexible career lifecycle approach to meet the needs of an increasingly diverse workforce and prevent burnout [50]. 

## Conclusions

It is not uncommon that the gravity of a problem is unnoticed until its adverse consequences are conspicuous. Physician burnout is an emerging scenario with similar dynamics. Chronic overwork, constant stressors, and a poor work environment cause emotional exhaustion, depersonalization, and a reduced sense of personal achievement. This can lead to poor patient care and follow-up, sub-optimal care increasing patient readmission rates, and poor physician-patient rapport, which can have adverse effects. Most importantly it leads to a decline in mental health, causing depression, substance abuse, and suicide among physicians, resulting in negative consequences for the hospital and the healthcare industry. In this review, we have discussed the impact of physician burnout causing professional and personal life compromise demeaning the purpose of life in totality. Burnout can be measured using various tools to assess the degree of severity, and hence, can be resolved at the budding stage by timely interventions by the governing heads of the hospitals. A focused approach with standard protocol must be laid down in healthcare provider institutions and offices. Professional coaching and stress reduction programs, which have shown to be effective in financial corporations, could be implemented in the healthcare industry. As team role implementation and cooperation among team members lead to less stress and reduced individual burden on performing tasks, social team building activities must be introduced to the work curriculum. Implementing hospital protocols for work duty division and structured roles are important factors to consider. Hospitals should provide paid leave to physicians along with time off to follow what they are particularly passionate about. Providing funding for team building activities will strengthen team relationships and also give employees a feeling that they are worthy and cared for. Strict following of ACGME work-hour restriction guidelines in all institutions will keep residents aware of their working hours and help them carry it forward once they are in the workforce as attending physicians. This will lead to developing a healthier generation of healthcare providers, better patient management and treatment outcomes, fewer errors and legal troubles, less financial burden, and an overall, a more efficient functioning in the healthcare sector.
